# Supplementary material for: Nanoparticle delivery of microRNA-146a regulates mechanotransduction in lung macrophages and mitigates injury during mechanical ventilation
Source: Nat Commun. 2021 Jan 12;12:289. doi: 10.1038/s41467-020-20449-w (PMC7804938; doi:10.1038/s41467-020-20449-w)
Supplement: Supplementary file 1 — Supplementary Information [file 41467_2020_20449_MOESM1_ESM.pdf]

SUPPLEMENTARY INFORMATION

Nanoparticle delivery of microRNA-146a regulates mechanotransduction in lung macrophages and mitigates lung injury during mechanical ventilation

Bobba and Fei et al

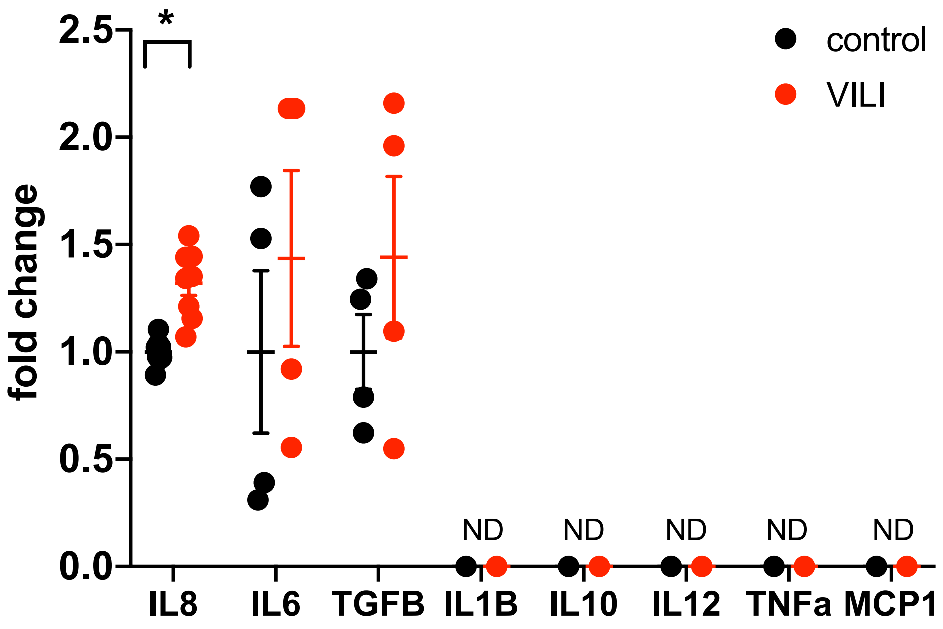

Supplementary Figure 1. Pressure induced secretion of cytokines/mediators in primary human alveolar macrophages subjected to oscillatory pressure (VILI) at an air-liquid interface in vitro. Data normally distributed, presented as mean $\pm$ SEM, p=0.0010 via two-tailed student's t-test comparing IL8 control and VILI groups. n=8 per group for IL8, all others n=4. *p<.05, ND=not detected.

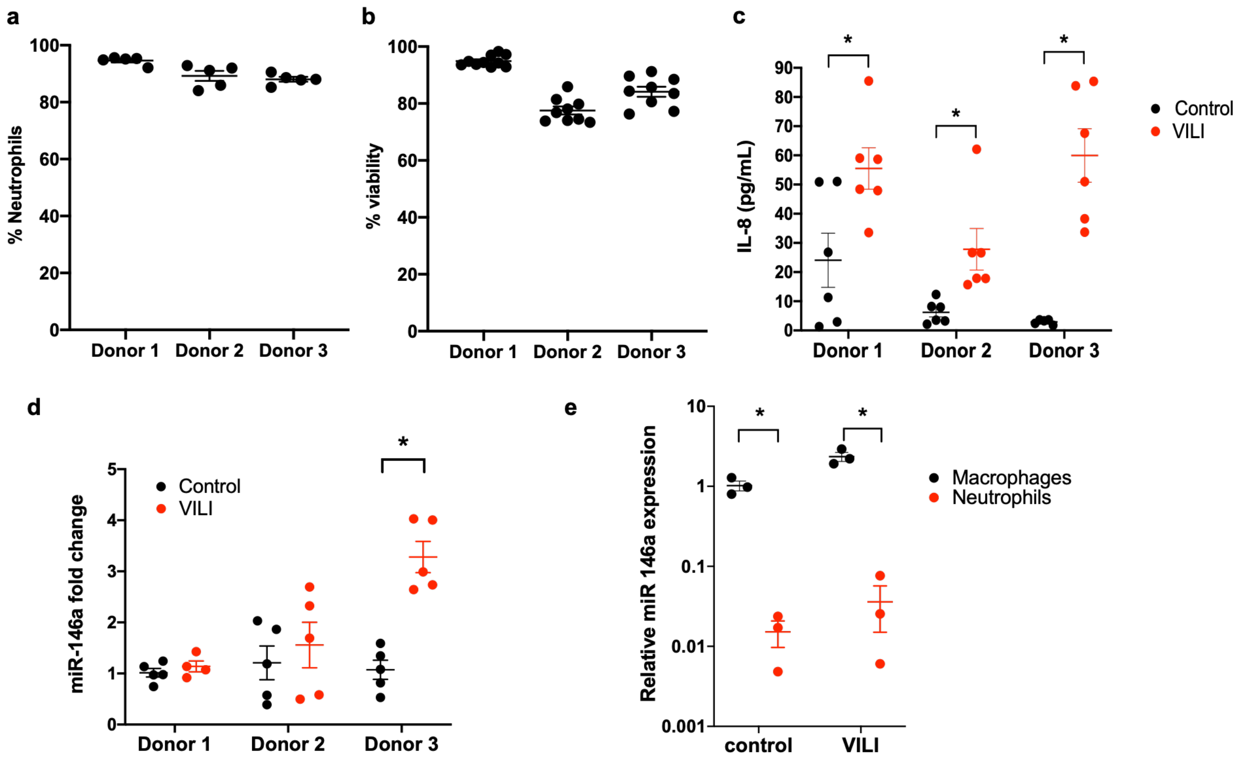

Supplementary Figure 2. Human neutrophils are mechanosensitive but do not consistently upregulate miR-146a following ventilator induced lung injury. **a** Percent neutrophils isolated from peripheral blood of 3 donors by cytospin staining. n=5 fields per donor. **b** Viability of neutrophils was assessed after culturing for 16 hours at air-liquid interface. n=10 fields for donor 1 and n=9 fields for donors 2 & 3 **c** IL8 secretion from primary human neutrophils from 3 donors subjected to 16 hours of oscillatory pressure at an air-liquid interface (VILI), compared to unpressurized controls. Data for donors 1 & 3 normally distributed, p=0.0226 for donor 1 and p=0.0003 for donor 3 via two-tailed student's t-test. Data for donor 2 log-normally distributed, p=0.0011 via two-tailed student's t-test on $\log_2$ transformed data. *p<0.05 compared to corresponding no pressure control, n=6 for each donor. **d** Relative miR-146a expression in neutrophils subjected to VILI, calculated by ΔΔCt method, normalized to unpressurized controls. Data normally distributed, p=0.0003 for donor 3 via two-tailed student's t-test. n=5 for each donor. **e** Relative mIR-146a expression in alveolar macrophages (from Figure 1) compared to neutrophils. p=0.0114 for control macrophage vs control neutrophil and p<0.0001 for VILI macrophage vs VILI neutrophil using Tukey's post-hoc comparison following 2-way ANOVA. Data are presented as mean ± SEM.

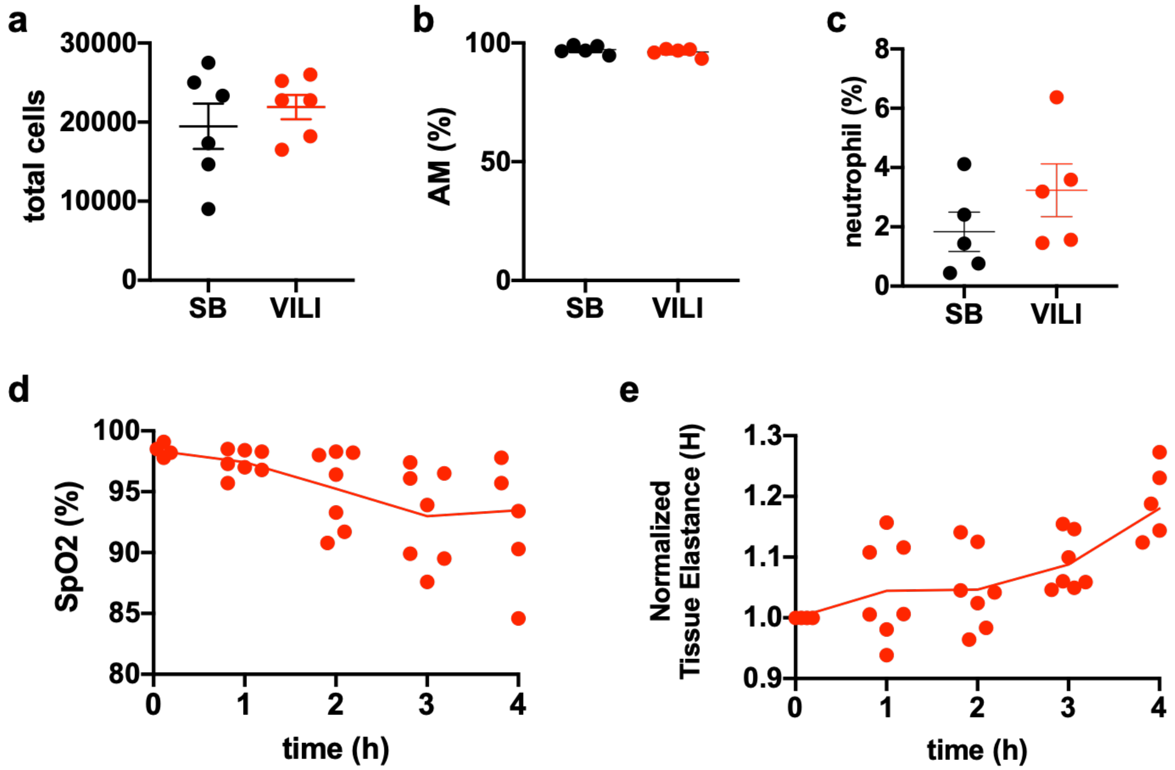

Supplementary Figure 3. Cell differential counts in spontaneous breathing and mechanically ventilated mice and lung physiologic data change during mechanical ventilation. a Total cell counts obtained from bronchoalveolar lavage (BAL) differential cell counts after ventilator induced lung injury (VILI). n=6 for spontaneously breathing (SB) and VILI groups. **b** Alveolar macrophage (AM) cell counts obtained from BAL following ventilation. n=5 per group. **c** Neutrophil cell counts obtained from BAL differential following ventilation. n=5 per group **d** Oxygenation throughout the duration of ventilation measured via pulse oximetry. n=7 per group. **e** Change in lung elastance throughout 4-hour period of mechanical ventilation. n=7 per group. All data presented as mean ± SEM

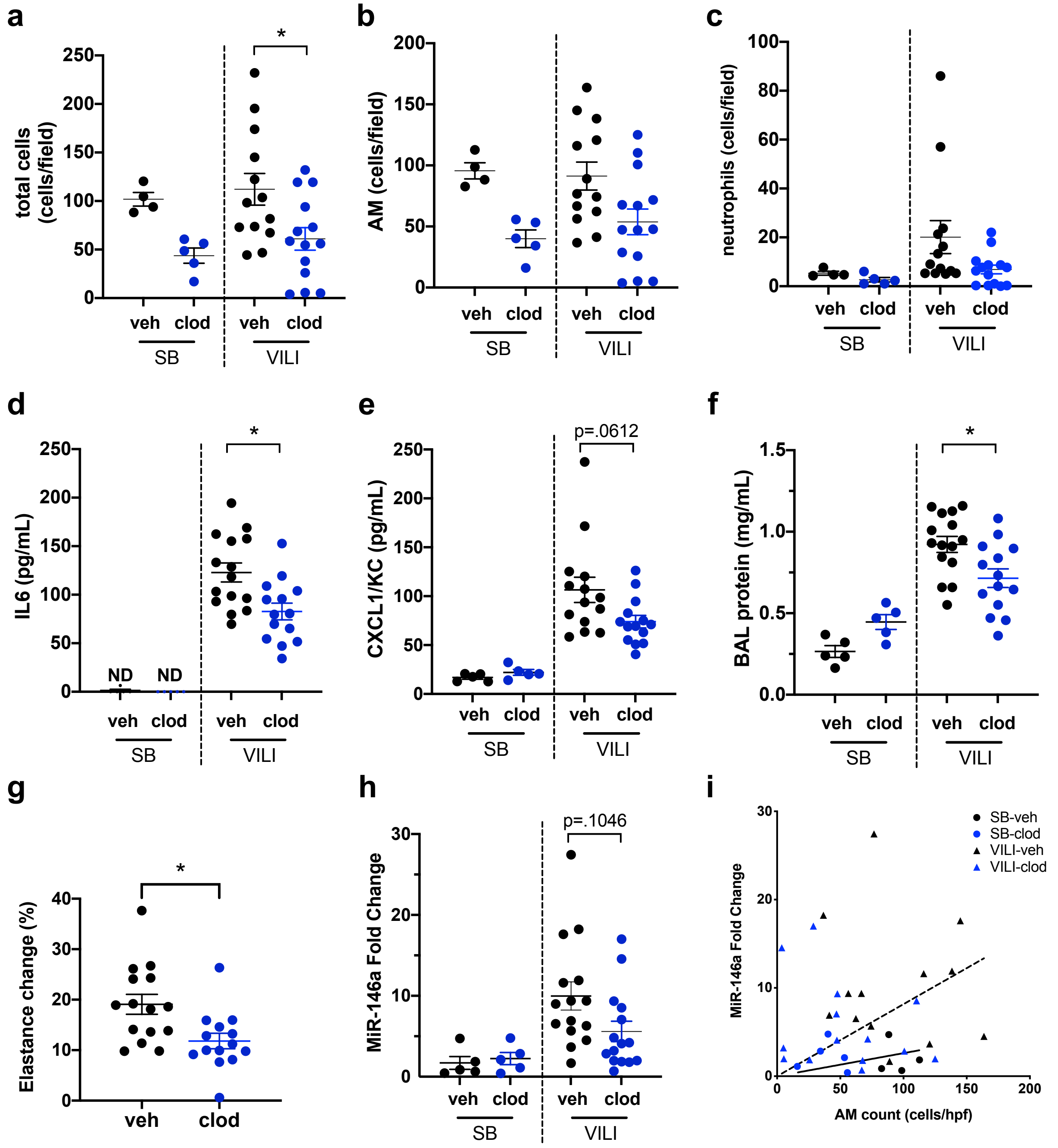

Supplementary Figure 4. Alveolar macrophage depletion reduces upregulation of miR-146a and dampens lung injury following injurious mechanical ventilation. a Total cell counts obtained from bronchoalveolar lavage (BAL) cytospins from spontaneously breathing (SB) and mechanically ventilated (VILI) groups following clodronate (clod) or vehicle (veh) treatment. Data normally distributed, p=0.0334 via Tukey post-hoc test following two-way ANOVA. n=4 veh-SB, n=5 clod-SB,n=13 veh-VILI, n=14 clod-VILI. **b** Alveolar macrophage (AM) cell counts obtained from BAL cytospin following ventilation. n=4 veh-SB, n=5 clod-SB,n=13 veh-VILI, n=14

clod-VILI. **c** Neutrophil cell counts obtained from BAL cytospin. n=4 veh-SB, n=5 clod-SB,n=13 veh-VILI, n=14 clod-VILI. **d** BAL IL6 from SB and mechanically ventilated (VILI) groups following clodronate or vehicle treatment. Data normally distributed, p=0.0070 via Tukey post-hoc test following two-way ANOVA. n= 5 SB groups & n=15 VILI groups. **e** BAL KC/CXCL1 from SB and VILI groups following clod or veh treatments. Data log-normally distributed, p=0.0612 via two-way ANOVA on $\log_2$ (fold change) with Tukey post-hoc test. n=5 SB groups, n=14 for veh-VILI, n=15 for clod-VILI. **f** BAL protein concentration from SB and VILI groups following clod or veh treatment. Data normally distributed, p=0.0212 via post-hoc Tukey test following two-way ANOVA. n= 5 SB groups & n=15 VILI groups. **g** Change in lung tissue elastance following ventilation, normalized to baseline elastance at initiation of ventilation. Data normally distributed, p=0.0077 via two-tailed student's t-test. n=15/group. **h** miR-146a levels from BAL cell RNA extracted following ventilation, calculated by ΔΔCt method, normalized to veh. Data log-normally distributed, p=0.1046 via two-way ANOVA on $\log_2$(fold change) with Tukey post-hoc test. n= 5 SB groups & n=15 VILI groups. **i** Correlation between AM cell count at miR-146a expression level. Solid line indicates linear regression with SB data only, p=0.0232, and dashed line indicates linear regression with VILI data only, p<0.0001. *p<0.05 for all other panels. Data are presented as mean ± SEM.

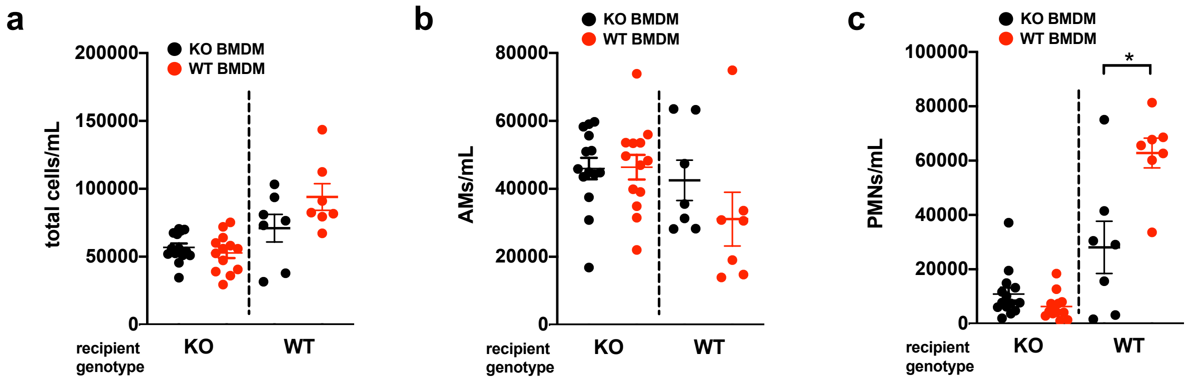

Supplementary Figure 5. Bronchoalveolar lavage total and differential cell counts from adoptive transfer experiments. a Total bronchoalveolar lavage cells from wild type (WT) or miR-146a knockout (KO) mice that received WT or KO bone marrow derived macrophages (BMDMs) prior to injurious ventilation. **b** BAL alveolar macrophages (AMs) in WT or KO recipients following injurious ventilation. **c** BAL neutrophils (PMNs) in WT or KO recipients following injurious ventilation. KO counts were log normally distributed, analyzed by two-tailed student's t-test on $\log_2$ transformed data. WT counts were normally distributed, p=0.0085 via two-tailed student's t-test. n=7/group for all WT recipients. n=14 for KO mice that received KO BMDMs and n=13 for KO mice that received WT BMDMs. *p<0.05. Data are presented as mean ± SEM.

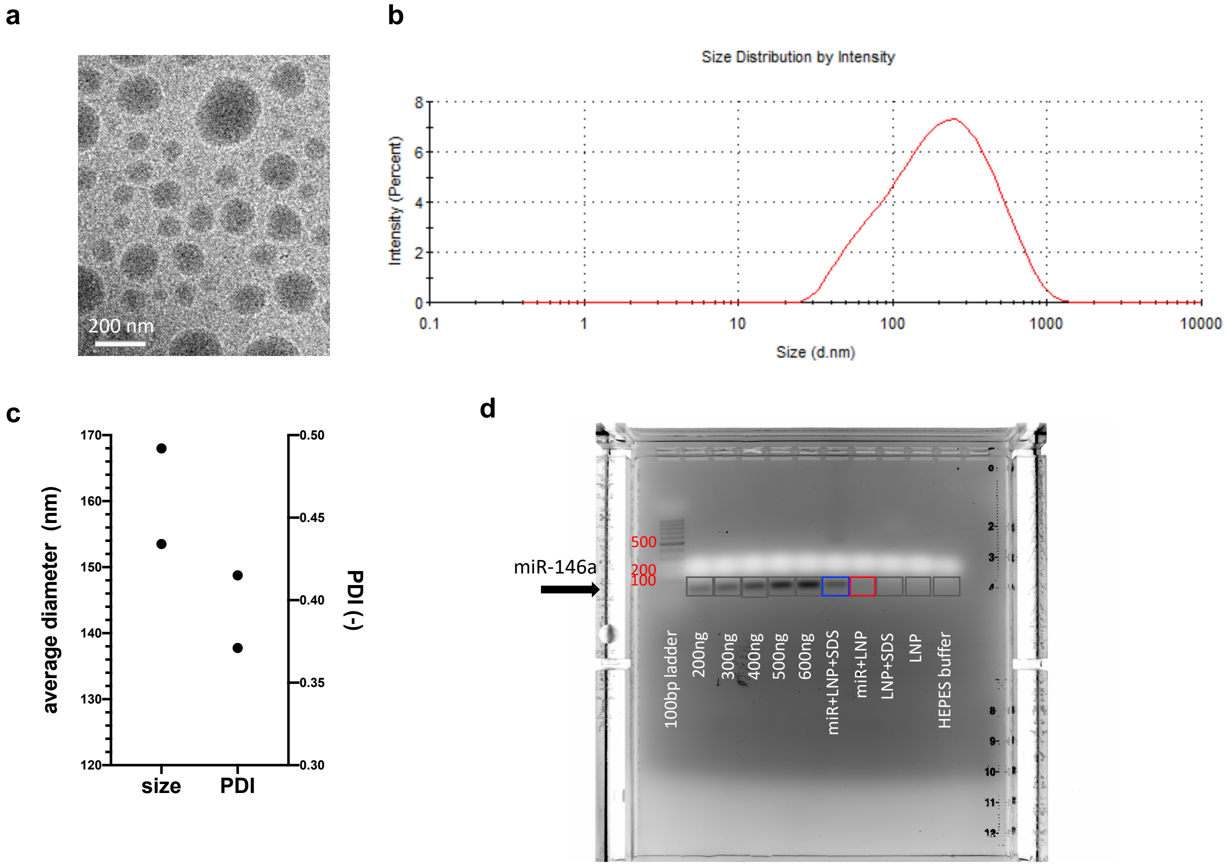

Supplementary Figure 6. Characterization of miR-146a loaded lipid nanoparticles. **a** Representative cryo-TEM image of miR-146a loaded lipid nanoparticles (LNPs). Experiment was performed three times. **b** Representative size distribution for a solution of miR loaded LNPs. Experiment was performed twice. **c** Size/diameter and polydispersity index (PDI) of LNPs measured by dynamic light scattering. n=2/group **d** Agarose gel electrophoresis demonstrating loading of miR-146a into the LNP. Boxes show bands for analysis where blue box indicates miR levels measured when LNPs were treated with sodium dodecyl sulfate (SDS)and red box indicates miR levels for intact LNPs.

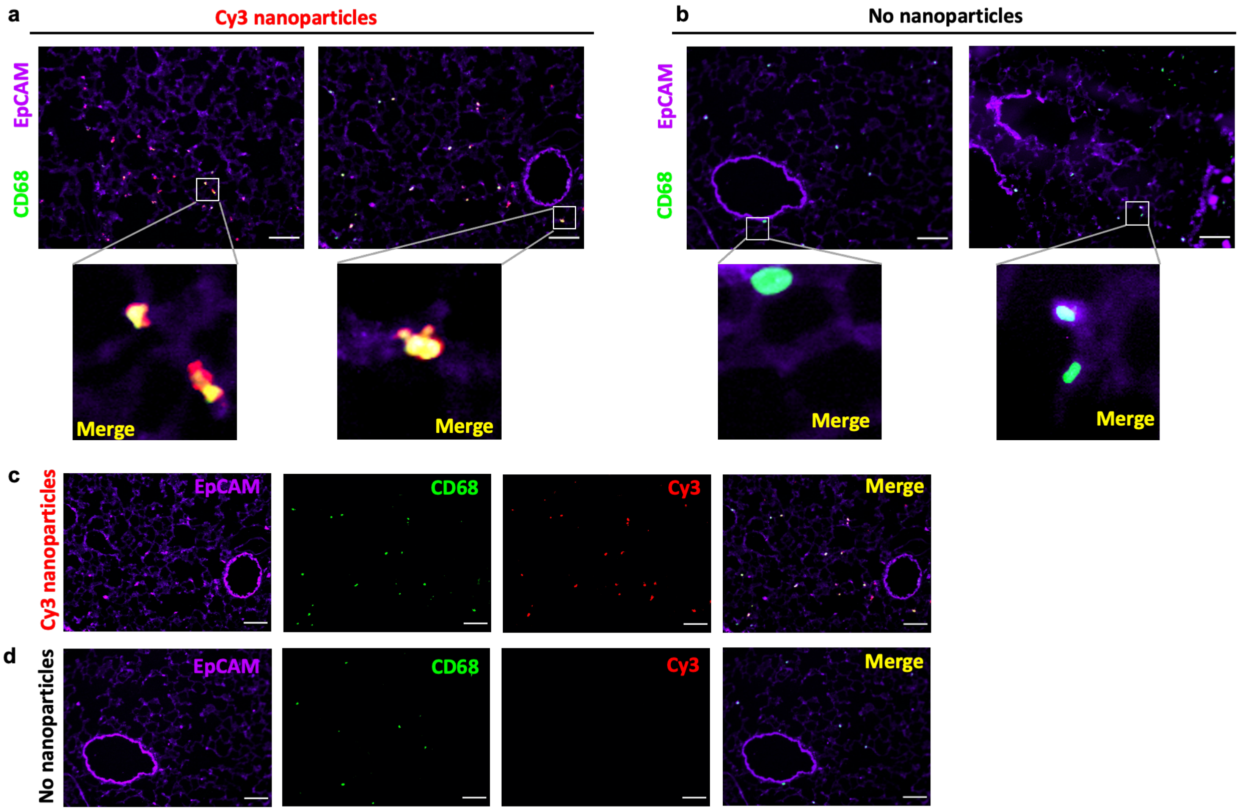

Supplementary Figure 7. Immunofluorescence imaging of lung tissue following Cy3 labeled lipid nanoparticle administration. a-b Representative images (10X magnification) of lung tissue from wild-type mice treated with Cy3 (red) labeled nanoparticles or control mice not treated with nanoparticles. Tissue was stained to identify macrophages (CD68, green) and epithelial cells (EpCAM, purple) **c-d** Additional images (10X) from nanoparticle treated mice and controls showing individual fluorescent channels. Experiment was performed twice. Scale bar: 100μm.

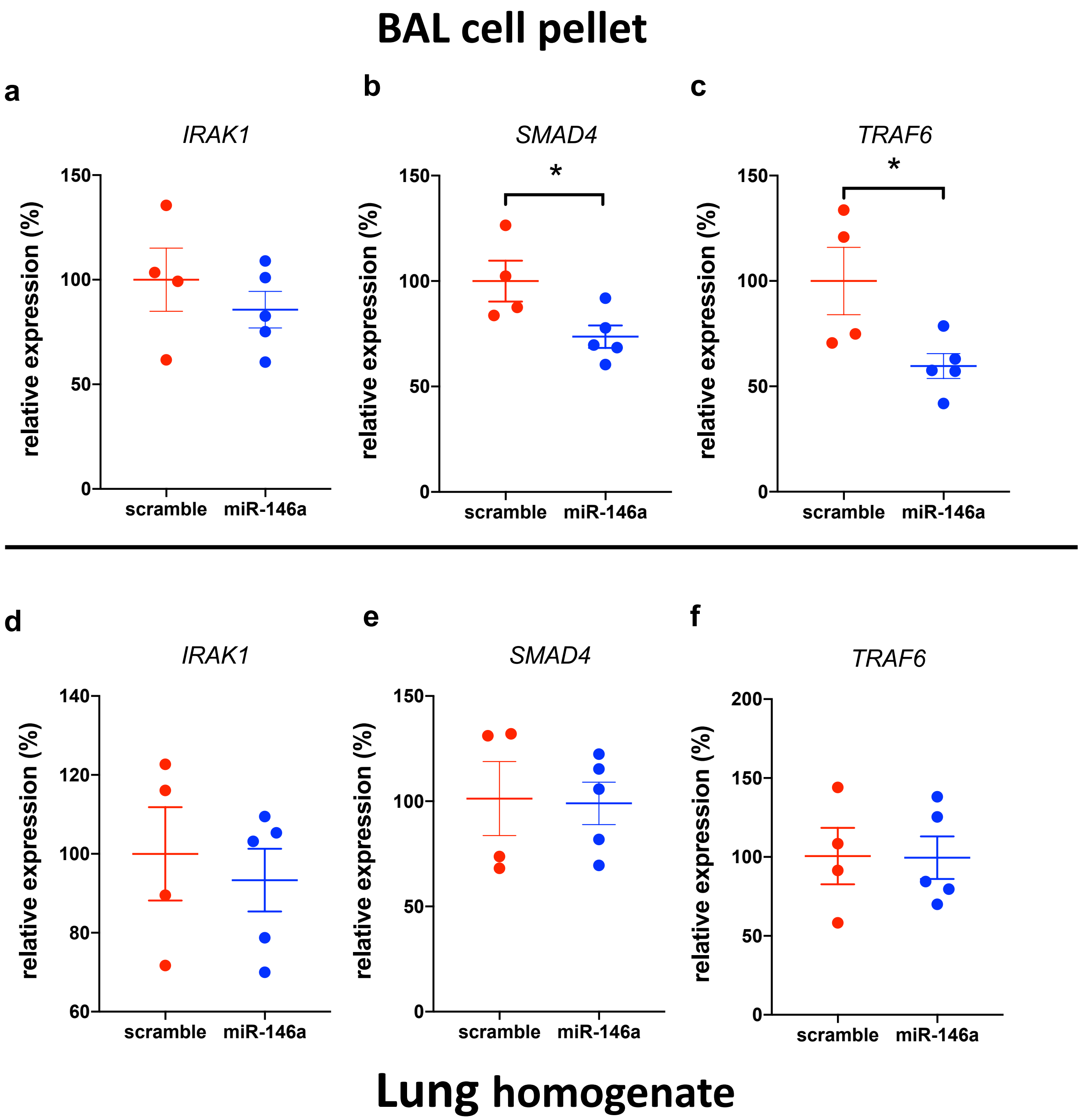

Supplementary Figure 8. miR-146a targets SMAD4 and TRAF6 are decreased in bronchoalveolar lavage cells of mice treated with miR-146a loaded lipid nanoparticles. mRNA levels of miR-146a targets IRAK1, SMAD4, and TRAF6 from total RNA extracted from bronchoalveolar lavage (BAL) cell pellets **(a-c)** and whole lung homogenate **(d-f)** of ventilated mice treated with scramble or miR-146a loaded nanoparticles. Expression calculated by ΔΔCt method and normalized to scramble. Data normally distributed. **b** p=0.0395 via student's t-test. **c** p=0.0356 via student's t-test on log_2(fold change). n=4 for scramble group and n=5 for miR group. Data presented as mean ± SEM.
