# Supplementary material for: Nanoparticle delivery of microRNA-146a regulates mechanotransduction in lung macrophages and mitigates injury during mechanical ventilation
Source: Nat Commun. 2021 Jan 12;12:289. doi: 10.1038/s41467-020-20449-w (PMC7804938; doi:10.1038/s41467-020-20449-w)
Supplement: Supplementary file 3 — Reporting Summary [file 41467_2020_20449_MOESM3_ESM.pdf]

nature research

Corresponding author(s): Joshua Englert

Last updated by author(s): 10/20/2020

Reporting Summary

Nature Research wishes to improve the reproducibility of the work that we publish. This form provides structure for consistency and transparency in reporting. For further information on Nature Research policies, see Authors & Referees and the Editorial Policy Checklist .

Statistics

For all statistical analyses, confirm that the following items are present in the figure legend, table legend, main text, or Methods section.

n/a	Confirmed	
☐	☒	The exact sample size (*n*) for each experimental group/condition, given as a discrete number and unit of measurement
☐	☒	A statement on whether measurements were taken from distinct samples or whether the same sample was measured repeatedly
☐	☒	The statistical test(s) used AND whether they are one- or two-sided<br>*Only common tests should be described solely by name; describe more complex techniques in the Methods section.*
☐	☒	A description of all covariates tested
☐	☒	A description of any assumptions or corrections, such as tests of normality and adjustment for multiple comparisons
☐	☒	A full description of the statistical parameters including central tendency (e.g. means) or other basic estimates (e.g. regression coefficient) AND variation (e.g. standard deviation) or associated estimates of uncertainty (e.g. confidence intervals)
☐	☒	For null hypothesis testing, the test statistic (e.g. *F*, *t*, *r*) with confidence intervals, effect sizes, degrees of freedom and *P* value noted<br>*Give P values as exact values whenever suitable.*
☒	☐	For Bayesian analysis, information on the choice of priors and Markov chain Monte Carlo settings
☒	☐	For hierarchical and complex designs, identification of the appropriate level for tests and full reporting of outcomes
☒	☐	Estimates of effect sizes (e.g. Cohen's *d*, Pearson's *r*), indicating how they were calculated

Our web collection on statistics for biologists contains articles on many of the points above.

Software and code

Policy information about availability of computer code

Data collection	Data was collected and recorded using Microsoft Excel (version 16.0) and as described in the Methods section.
Data analysis	Software used to analyze data: Graphpad Prism 8, SAS version 9.4, Fiji (version 1.51), MATLAB (version R2018b)

For manuscripts utilizing custom algorithms or software that are central to the research but not yet described in published literature, software must be made available to editors/reviewers. We strongly encourage code deposition in a community repository (e.g. GitHub). See the Nature Research guidelines for submitting code & software for further information.

Data

Policy information about availability of data

All manuscripts must include a data availability statement. This statement should provide the following information, where applicable:

- Accession codes, unique identifiers, or web links for publicly available datasets
- A list of figures that have associated raw data
- A description of any restrictions on data availability

The data that support the findings of this study are available from the corresponding authors upon reasonable request. Source data are provided with this paper.

Field-specific reporting

Please select the one below that is the best fit for your research. If you are not sure, read the appropriate sections before making your selection.

☒ Life sciences ☐ Behavioural & social sciences ☐ Ecological, evolutionary & environmental sciences

For a reference copy of the document with all sections, see nature.com/documents/nr-reporting-summary-flat.pdf

Life sciences study design

All studies must disclose on these points even when the disclosure is negative.

Sample size	Experimental sample size was determined by performing a pilot experiment with 6 animals and the sample mean and standard deviation estimates were used to calculate the final sample size with power of 0.8 and α = .05. Sample size calculations were not performed in vitro studies and sample sizes were estimated based on prior experiments in our laboratory and previously published data.
Data exclusions	Outlier testing was performed to identify statistical outliers using non-linear regression via the ROUT method with a Q threshold of 1% for all experiments. Outlier testing was performed prior to statistical testing for differences between groups and outliers were excluded from statistical testing for differences between groups. Outliers was detected in Figures 3B, 3C, 3E, 4A, 4B, 4F, 6D, 6E, 6F, 6G, 6N, Supp Fig 2C-D, Supp Fig 4D, Supp Fig 4E, Supp Fig 4F, Supp Fig 4G. Complete data sets including outliers can be found in the Source Data File.
Replication	The nanoparticle encapsulation experiment requested by reviewers was only performed once (Supplemental Figure 6D). All other in vitro experiments were replicated and attempts at replication were successful. Animal studies were not replicated given that sample sizes were determined a priori based on power calculations.
Randomization	Animal order of ventilation studies was randomized for all experiments. For in vitro experiments, cells were randomly assigned to treatment groups.
Blinding	Blinding was not performed in data acquisition or analysis. Experiments required pre-treatment with specific agents (clodronate, nanoparticles, etc), and only CB was trained in both pre-treatment and experimental protocols.

Reporting for specific materials, systems and methods

We require information from authors about some types of materials, experimental systems and methods used in many studies. Here, indicate whether each material, system or method listed is relevant to your study. If you are not sure if a list item applies to your research, read the appropriate section before selecting a response.

Materials & experimental systems

n/a	Involved in the study	
☐	☒	Antibodies
☐	☒	Eukaryotic cell lines
☒	☐	Palaeontology
☐	☒	Animals and other organisms
☐	☒	Human research participants
☒	☐	Clinical data

Methods

n/a	Involved in the study	
☒	☐	ChIP-seq
☒	☐	Flow cytometry
☒	☐	MRI-based neuroimaging

Antibodies

Antibodies used	Antibodies from abcam: 1. Rabbit anti-EpCAM (ab237385), 2. Rat anti-CD68 (ab201844).
Validation	All antibodies were obtained from commercially available sources and validated by the manufacturer for the application used. Anti-EpCAM (ab237385) antibody is validated for ICC/IF and it reacts with mouse protein. Anti-CD68 (ab201844) antibody is validated for ICC/IF and it reacts with mouse protein. Both antibodies stain positive on the positive cell line (4T1) and show no stain on the negative cell line (NIH-3T3) per the manufacturer.

Eukaryotic cell lines

Policy information about cell lines

Cell line source(s)	THP-1 (ATCC TIB-202)
Authentication	Cell line was not authenticated
Mycoplasma contamination	Cell line not tested for mycoplasma
Commonly misidentified lines (See ICLAC register)	No commonly misidentified cell lines were used in our studies.

Animals and other organisms

Policy information about studies involving animals; ARRIVE guidelines recommended for reporting animal research

Laboratory animals	All laboratory mice were purchased from Jackson lab<br>1. MiR-146a knock out mice, stock: 016239<br>2. C57BL/6J wild-type, stock: 000664<br><br>Manuscript describes all aspects of ARRIVE guidelines for reporting animal experiments. Additional detailed information such as housing, husbandry, adverse events, and baseline data available from corresponding author upon request.
Wild animals	Study did not involve wild animals
Field-collected samples	No field collected samples were used
Ethics oversight	Ohio State Institutional Animal Care and Use Committee approved animal experiments and periodically inspected lab for IACUC compliance. IACUC protocols: 2011A00000081-R2 and 2013A00000105-R1.

Note that full information on the approval of the study protocol must also be provided in the manuscript.

Human research participants

Policy information about studies involving human research participants

Population characteristics	Relevant population characteristics are outlined in table 1 of the manuscript. These include: age, gender, race, white blood cell count, lactate concentration, use of vasopressors, SIRS score, systolic BP and BAL cell count, diagnosis with ARDS and use of mechanical ventilation. Methods section describes all relevant tier 1 characteristics recommended by BRISQ reporting guidelines.
Recruitment	Potential subjects that were undergoing a clinically indicated bronchoscopy for suspected infection were identified from the medical intensive care unit and pulmonary consult services. All patients 18 years of age or older were considered eligible. The patient or his/her legal authorized representative was approached by one of the investigators for participation in the study. Given that the decision to proceed with bronchoscopy was at the discretion of the treating providers, it is possible that patients with mild or atypical infections may not have been included which could introduce potential selection bias. We believe that it is unlikely that this impacted our results since our primary comparison was to analyze differences in miR-146a levels between spontaneously breathing and mechanically ventilated patients.
Ethics oversight	The Ohio State Institutional Review board approved of sample collection (IRB protocols: 2016H0009 & 2011H0059)

Note that full information on the approval of the study protocol must also be provided in the manuscript.
